# Multifaceted neuroprotective approach of Trolox in Alzheimer's disease mouse model: targeting Aβ pathology, neuroinflammation, oxidative stress, and synaptic dysfunction

**DOI:** 10.3389/fncel.2024.1453038

**Published:** 2024-09-17

**Authors:** Muhammad Tahir, Min Hwa Kang, Tae Ju Park, Jawad Ali, Kyonghwan Choe, Jun Sung Park, Myeong Ok Kim

**Affiliations:** ^1^Division of Life Science and Applied Life Science (BK21 FOUR), College of Natural Sciences, Gyeongsang National University, Jinju-si, Republic of Korea; ^2^Haemato-Oncology/Systems Medicine Group, Paul O'Gorman Leukaemia Research Centre, Institute of Cancer Sciences, MVLS, University of Glasgow, Glasgow, United Kingdom; ^3^Department of Psychiatry and Neuropsychology, School for Mental Health and Neuroscience (MHeNs), Maastricht University, Maastricht, Netherlands; ^4^Alz-Dementia Korea Co., Jinju-si, Republic of Korea

**Keywords:** Alzheimer's disease (AD), amyloid beta plaques (Aβ), neurofibrillary tangles (NFTs), Trolox, neuroinflammation, oxidative stress and neurodegeneration

## Abstract

Alzheimer's disease (AD) is a progressive neurodegenerative disorder pathologically characterized by the deposition of amyloid beta (Aβ) plaques and neurofibrillary tangles (NFTs) in the brain. The accumulation of these aggregated proteins causes memory and synaptic dysfunction, neuroinflammation, and oxidative stress. This research study is significant as it aims to assess the neuroprotective properties of vitamin E (VE) analog Trolox in an Aβ_1 − 42_-induced AD mouse model. Aβ_1 − 42_ 5μL/5min/mouse was injected intracerebroventricularly (i.c.v.) into wild-type adult mice brain to induce AD-like neurotoxicity. For biochemical analysis, Western blotting and confocal microscopy were performed. Remarkably, intraperitoneal (i.p.) treatment of Trolox (30 mg/kg/mouse for 2 weeks) reduced the AD pathology by reducing the expression of Aβ, phosphorylated tau (p-tau), and β-site amyloid precursor protein cleaving enzyme1 (BACE1) in both cortex and hippocampus regions of mice brain. Furthermore, Trolox-treatment decreased neuroinflammation by inhibiting Toll-like receptor 4 (TLR4), phosphorylated nuclear factor-κB (pNF-κB) and interleukin-1β (IL-1β), and other inflammatory biomarkers of glial cells [ionized calcium-binding adaptor molecule 1 (Iba1) and glial fibrillary acidic protein (GFAP)]. Moreover, Trolox reduced oxidative stress by enhancing the expression of nuclear factor erythroid-related factor 2 (NRF2) and heme oxygenase 1 (HO1). Similarly, Trolox-induced synaptic markers, including synaptosomal associated protein 23 (SNAP23), synaptophysin (SYN), and post-synaptic density protein 95 (PSD-95), and memory functions in AD mice. Our findings could provide a useful and novel strategy for investigating new medications to treat AD-associated neurodegenerative diseases.

## 1 Introduction

Alzheimer's disease (AD) is the primary contributor to the onset of dementia, a devastating neurodegenerative disorder categorized by the growing loss of cholinergic neurons (Godoy et al., 2014; Stefanova et al., 2015). This neurodegeneration results in a decline in cognitive abilities, typically evident through changes in thinking, learning, memory, and daily life activities (Denny et al., 2021). Despite extensive research, the exact cause of AD remains unknown. The two pathological hallmarks of AD, first categorized by Alois Alzheimer, are the deposition of amyloid plaques and neurofibrillary tangles (NFTs) in the female patient (Auguste D) brain having the age of 51 years old. The two transmembrane enzymes (β-site amyloid precursor protein cleaving enzyme1 (BACE1) and α-secretase) undergo proteolysis of amyloid precursor protein (APP) into amyloid β-peptides (Aβ_1 − 42_). On the other side, hyperphosphorylation of the microtubule-associated tau protein leads to forming NFTs of varying lengths and shapes. The deposition of Aβ_1 − 42_ peptides and NFTs in the brain leads to neuroinflammation, oxidative stress, and synaptic and memory dysfunctions, followed by neurodegeneration (Miranda et al., 2000; Behl et al., 1994; Koppal et al., 1998; Leissring et al., 2002; Schieber and Chandel, 2014; Badshah et al., 2019). Both the cytotoxic proteins (Aβ_1 − 42_ peptides and p-Tau) in the brain trigger immune responses through glial cells, specifically microglia and astrocytes, leading to subsequent neuroinflammation and release different proinflammatory mediators and cytokines (phosphorylated nuclear factor kappa B, interleukin 1β and Toll-like receptor 4). The neurotoxic effect of Aβ_1 − 42_ has been observed and supported by different *in vitro* studies that are mediated by free radical mechanisms (Miranda et al., 2000; Behl et al., 1994; Koppal et al., 1998) and variation in calcium ion (Ca^2+^) homeostasis in neuronal cells (Leissring et al., 2002). In addition, AD progression involves oxidative stress induced by reactive oxygen species (ROS), which are free radical molecules produced abnormally during cellular metabolism. This oxidative stress is associated with cellular damage, affecting DNA, proteins, and lipids, contributing to various diseases such as neurodegenerative disorders, cardiovascular diseases, and cancer (Schieber and Chandel, 2014; Badshah et al., 2019; Agostinho and Oliveira, 2010). Under natural conditions, reactive antioxidants develop in a biologically controlled environment, impacting cellular processes such as autophagy, inflammation, immunological response, and cell division. Some of the significant antioxidant genes, including nuclear factor erythroid-related factor 2 (NRF2), have a detrimental effect and regulate neuroinflammation, oxidative stress, and synapse formation in the brain (Khan et al., 2018; Huang et al., 2005). Numerous studies have proposed that enhanced oxidative stress in neuronal cells disrupts the internal antioxidant system, leading to downregulation of NRF2 and its target gene heme oxygenase 1 (HO1) protein expression levels (Giordano et al., 2020; Khan et al., 2019).

A growing number of plant-derived natural products are being investigated as therapeutic agents for preventing and treating neurological disorders such as AD and Parkinson's disease (Huang and Mucke, 2012). Vitamins are essential micronutrients for strengthening the immune system and fighting infections. They contribute to neurogenesis, the defense mechanism of neurons, and are involved in metabolic reactions, neuron survival, and transmission (Kumar et al., 2022). Additionally, vitamin E (VE) is recognized as a vital micronutrient in the diet of most animals. It comprises tocopherols and tocotrienols, with α-tocopherol being the most potent homolog (Xu et al., 2023). Several previous studies have revealed that VE performs various functions, such as improving animal growth and reproduction, sustaining cell membrane homeostasis, and enhancing body immunity and anti-inflammatory and antioxidant capabilities (Rengaraj and Hong, 2015; El-Sayed and Izquierdo, 2022; Sattler et al., 2003; Kim et al., 2017). In this study, Trolox (6-hydroxy-2, 5, 7, 8-tetramethylchroman-2-carboxylic acid), a hydrophilic counterpart to α-tocopherol and a prominent form of tocopherols in the human body, was employed against Aβ_1 − 42_-induced AD mouse model (Massey and Burton, 1990; Lúcio et al., 2009). Trolox, like α-tocopherol, acts as a potent lipid antioxidant by scavenging lipid peroxyl free radicals, preventing peroxidation of polyunsaturated fatty acids, and protecting cell membranes from oxidative damage. Unlike α-tocopherol, Trolox is water-soluble and lipophilic, reaching both water and lipid segments of cells. It has become more significant than α-tocopherol in several biochemical assays due to its superior radical scavenging efficacy against peroxyl and alkoxyl radicals (Giordano et al., 2020). Trolox has been previously reported as a potent antioxidant and anti-inflammatory against 1-methyl-4-phenyl-1,2,3,6-tetrahydropyridine (MPTP)-induced Parkinson's disease in a mouse model (Atiq et al., 2023), a potent antioxidant and inflammatory candidate that alleviates inflammation and oxidative stress in human and murine primary alveolar type II cells from injury (Messier et al., 2013) and preventing oxidative stress-induced apoptosis in mouse thymocytes (Forrest et al., 1994), and renal normal rat kidney 52e cells) (Guo et al., 2012). At present, we hypothesized that Trolox might rescue mice brains from the Aβ_1 − 42_-induced neuroinflammation mediated neurodegeneration and oxidative stress in the Aβ_1 − 42_-induced AD mouse model. Different neuroprotective properties, including antioxidant, anti-inflammatory, and synaptic properties, were assessed by biochemical, cognitive, and immunohistochemical assays.

## 2 Materials and methods

### 2.1 Animals

Wild-type male mice (C57BL/6N *n* = 32, 8 weeks old, 25–30 g body weight) were purchased from Samtako Biolabs (Ulsan, South Korea). Under a 12-h light/dark cycle, all mice were housed for habituation for 7 days in temperature- and humidity-controlled settings with free access to food and water. All the experimental protocols for animal care and treatment were approved (approval ID: 125, animal ethics code: GNU-200331-M0020) by the Animal Ethics Committee of the Division of Applied Life Sciences, Department of Biology, Gyeongsang National University, South Korea.

### 2.2 Drug treatment with animal groupings

**Figure 1:**
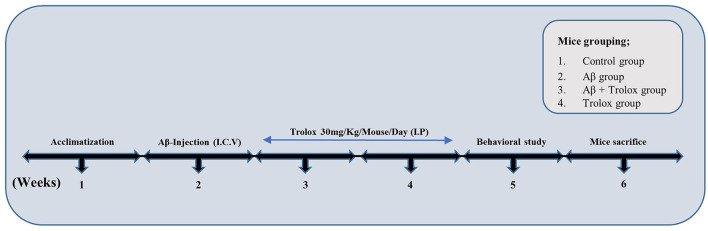
Experimental plan of Trolox against Aβ_1 − 42_-induced Alzheimer's disease mouse model. The experimental paradigm consisted of 6 weeks. Mice were acclimatized in the first week, Aβ-Intracerebroventricular (i.c.v.) injection was administered in the second week, and Trolox (30 mg) drug was treated in the third and fourth weeks. Behavioral analysis (MWM, Y-maze) was performed on the fifth week, and the mice were euthanized on the sixth week.

A sterile saline solution was used as a solvent to prepare a stock solution of human-derived Aβ_1 − 42_ peptide (Cat. ^#^PP69, Sigma–Aldrich, St. Louis, MO, USA) at 1 mg/ml concentration. This solution was then incubated at 37°C for 4 days to allow appropriate accumulation of Aβ_1 − 42_ oligomers. Mice were anesthetized with a combination of Rompun (xylazine, 10 mg/kg) and Zoletil (ketamine, 90 mg/kg) dissolved in 0.9% saline and injected intraperitoneally (i.p.). The Aβ_1 − 42_ peptide or vehicle (5 μL or 0.9% NaCl/5 min/mouse) was stereotaxically injected intracerebroventricular (i.c.v.) into the mice brain ventricles by using a Hamilton microsyringe in different measurements of −2.4 mm dorsoventral (DV), −0.2-mm anteroposterior (AP) and 1-mm mediolateral (ML) to the bregma point. The stereotaxic surgical procedure was conducted in a room equipped with a temperature control system to maintain the animals' body temperature between 36°C and 37°C. The body temperature was regularly monitored with a thermometer due to the hypothermic effects of anesthesia, which can induce tau phosphorylation (Ali et al., 2015). The animals were assigned into four groups: (1) control group (0.9% saline as control mice), (2) Aβ_1 − 42_ group (5 μL/5 min/mouse), (3) Aβ_1 − 42_ + Trolox group [30 mg/kg (i.p.) per day for 2 weeks] and (4) Trolox alone group as a sham group [30 mg/kg (i.p.) per day for 2 weeks, dissolved in phosphate buffer saline (pH 7.4) (Figure 1)]. The dose of Trolox was selected as reported previously (Sharma and Sayyed, 2006). Each group of animals contained eight mice, and each of them was allocated for morphological study and Western blot analysis.

### 2.3 Behavioral study

#### 2.3.1 Y-maze test

The Y-maze was performed as described previously (Ikram et al., 2019). The translucent plexiglass sheets made up of the Y-maze apparatus were each 50 cm long, 20 cm high, and 10 cm wide at the bottom and top. The mice were kept in the middle of the instrument for 8-min phases, allowing free movement to explore the maze. The consecutive entry of the mice into the three arms in an overlapping triplet was detected as one of the spontaneous changes. Spontaneous alternation behavior in the form of higher percentage values was considered to improve cognitive capabilities. The alteration behavior has been presented in percentage using the formula: (entries into three arms consecutively/total number of arm entries – 2) × 100.

#### 2.3.2 Morris water maze test

The cognitive behavior of mice was observed by distributing mice into four groups, *n* = 8 for each group. The Morris water maze (MWM) was performed as previously described (Yan and Vassar, 2014; Vassar, 2014). MWM consisted of a circular tank (100 cm in diameter, 40 cm in height) containing water (23 ± 1°C) with a depth of 15.5 cm, and a non-toxic white-colored ink was dissolved by making transparent water opaque. The transparent escape platform was kept in the center of one quadrant 1 cm below the water's surface to make it invisible. It measures 10 cm in diameter and 20 cm in height. Each mouse was trained once daily for 5 days consecutively on a single hidden platform in one of the three rotating quadrants. For the valuation of memory, a probe test was conducted the next day of training by removing the hidden platform, and mice were allowed to swim freely for the time scale of 60 s. The number of crossovers where the hidden platform was present during the training session and the time spent in the target quadrant were recorded. A video-recording software (SMART Pan lab, Harvard Apparatus, Holliston, MA, USA) was used for data recording purposes.

### 2.4 Protein extraction

The protein extraction was performed as previously described (Chuang et al., 2017). Using ketamine and xylazine, the mice were anesthetized intramuscularly (i.m.), euthanized, and removed. The brain, the cortex, and hippocampus regions were carefully dissected and were preserved at −80°C for further experiments. The cortex and hippocampus regions were homogenized in PRO-PREP^TM^ extraction solution (iNtRON Biotechnology, Inc., Sungnam, South Korea) and centrifuged at 13,000 *g* for 30 min at 4°C. After centrifugation, the supernatant was collected and kept at −80°C for the Western blot analysis.

### 2.5 Western blot analysis

The immunoblotting analysis followed established protocols from previous studies (Khan et al., 2021). Briefly, a Bio-Rad assay kit (Bio-Rad Laboratories, Irvine, CA, USA) was used to determine protein concentrations. Proteins extracted from the brains of all experimental mice groups in equal amounts were subjected to SDS-PAGE on 4–15% gels with a prestained protein marker (GangNam-STAIN, iNtRON Biotechnology, Dallas, TX, USA) and then transferred to polyvinylidene difluoride (PVDF) membranes (Immobilon-PSQ, Transfer membrane, Merck Millipore, Burlington, MA, USA). After PVDF membrane transfer, all membranes were blocked with 5% skim milk (Difco™ Skim Milk, BD, France) and then incubated with primary antibodies overnight at 4°C. Furthermore, after incubation, the membranes were probed with horseradish peroxidase (HRP)-conjugated secondary antibodies. The protein bands were detected by applying an Enhanced chemiluminescent (ECL) detecting solution (ATTO Corporation, Tokyo, Japan), further scanning the X-ray films. A densitometric analysis of the protein bands was performed using ImageJ software (v.1.50, NIH, Bethesda, MD, USA).

### 2.6 Sample preparation for morphological analysis of brain specimens

The brain specimens for morphological examination were prepared as described previously (Khan et al., 2023). All the mice were anesthetized (ketamine and xylazine) (i.m.) and transcardially perfused with 0.9% normal saline and 4% paraformaldehyde solution. The brains were carefully removed and fixed in ice–cold 4% neutral buffer paraformaldehyde at 4°C for 72 h, and then all the brains were dehydrated in 20% sucrose for 72 h. Furthermore, the brains were placed in an optimum cutting temperature (OCT) compound obtained from Finetek Japan Co., Ltd., Tokyo, Japan, and then frozen. Brain sections of 14 μm were obtained on gelatin-coated slides using a microtome (CM 3050C cryostat, Leica, Germany).

### 2.7 Immunofluorescence staining

Immunofluorescence analysis was carried out as conducted previously (Amin et al., 2017). All slides were washed twice for 10 min with phosphate-buffered saline (PBS, 1%). The slides were then incubated at room temperature for 5 min with proteinase K. A blocking solution containing 0.3% Triton X-100 and 2% normal serum dissolved in 1% PBS was applied to each slide for 1 h after washing. All slides were treated with primary antibodies overnight at 4°C. Following incubation, brain sections were washed with PBS and exposed to secondary antibodies that were tetramethylrhodamine isothiocyanate (TRITC) or fluorescein isothiocyanate (FITC) (antirabbit, antigoat, or antimouse) diluted 1:50 in PBS for 90 min at room temperature. After secondary antibody treatment, the tissue slides were counterstained for 8 min with 4′,6-diamidino-2-phenylindole (DAPI) nucleus solution. Then, the slides were covered with a coverslip using a mounting media by applying DPX (Distyrene Plasticizer Xylene). Immunofluorescence imaging was performed using a confocal laser scanning microscope (FV 1000MPE, Olympus, Japan).

### 2.8 Antibodies

Table 1 The primary antibodies used in this study.

**Table 1:** Antibodies used for immunofluorescence (IF) and Western blot (WB) analysis.

**Protein targets**	**Host**	**Application**	**Manufacturer**	**Catalog number**	**Concentration**
HO1	Mouse	WB	Santa Cruz Biotechnology	SC 136961	1:1,000
NRF2	Mouse	WB/IF	Santa Cruz Biotechnology	SC 365949	1:1,000/1:100
Aβ	Mouse	WB/IF	Santa Cruz Biotechnology	SC 28365	1:1,000/1:100
p-tau	Mouse	WB/IF	Santa Cruz Biotechnology	SC 32275	1:1,000/1:100
BACE1	Mouse	WB	Santa Cruz Biotechnology	SC 33711	1:1,000
GFAP	Mouse	WB/IF	Santa Cruz Biotechnology	SC 33673	1:1,000/1:100
Iba1	Mouse	WB/IF	Santa Cruz Biotechnology	SC 398406	1:1,000/1:100
TLR4	Mouse	WB	Santa Cruz Biotechnology	SC 293072	1:1,000
IL-1β	Mouse	WB	Santa Cruz Biotechnology	SC 32294	1:1,000
pNF-kB	Mouse	WB	Santa Cruz Biotechnology	SC 136548	1:1,000
SNAP23	Mouse	WB	Santa Cruz Biotechnology	SC 374215	1:1,000
SYN	Mouse	WB	Santa Cruz Biotechnology	SC 17750	1:1,000
PSD-95	Mouse	WB	Santa Cruz Biotechnology	SC 71933	1:1,000
β-Actin	Mouse	WB	Santa Cruz Biotechnology	SC 47778	1:1,000

## 3 Statistical data analysis

The reported result data was analyzed as mean ± standard error of the mean (SEM) using one-way ANOVA followed by Tukey's test for comparing various treatment groups of mice for the replicate of three experiments. For this purpose, eight mice/group were taken, and the *p*-value (*p* < 0.05) was set as a standard for the significant differences among the groups. Significance: ^#^*p* ≤ 0.05, ^*##*^*p* ≤ 0.01, and ^*###*^*p* ≤ 0.001; ^*^p ≤ 0.05, ^**^p ≤ 0.01, and ^***^p ≤ 0.001. ImageJ software was used for the immunohistological quantitative study which is shown as arbitrary units (AU).

## 4 Results

### 4.1 Trolox-treatment reversed Aβ_1 − 42_-induced cognitive impairment

**Figure 2:**
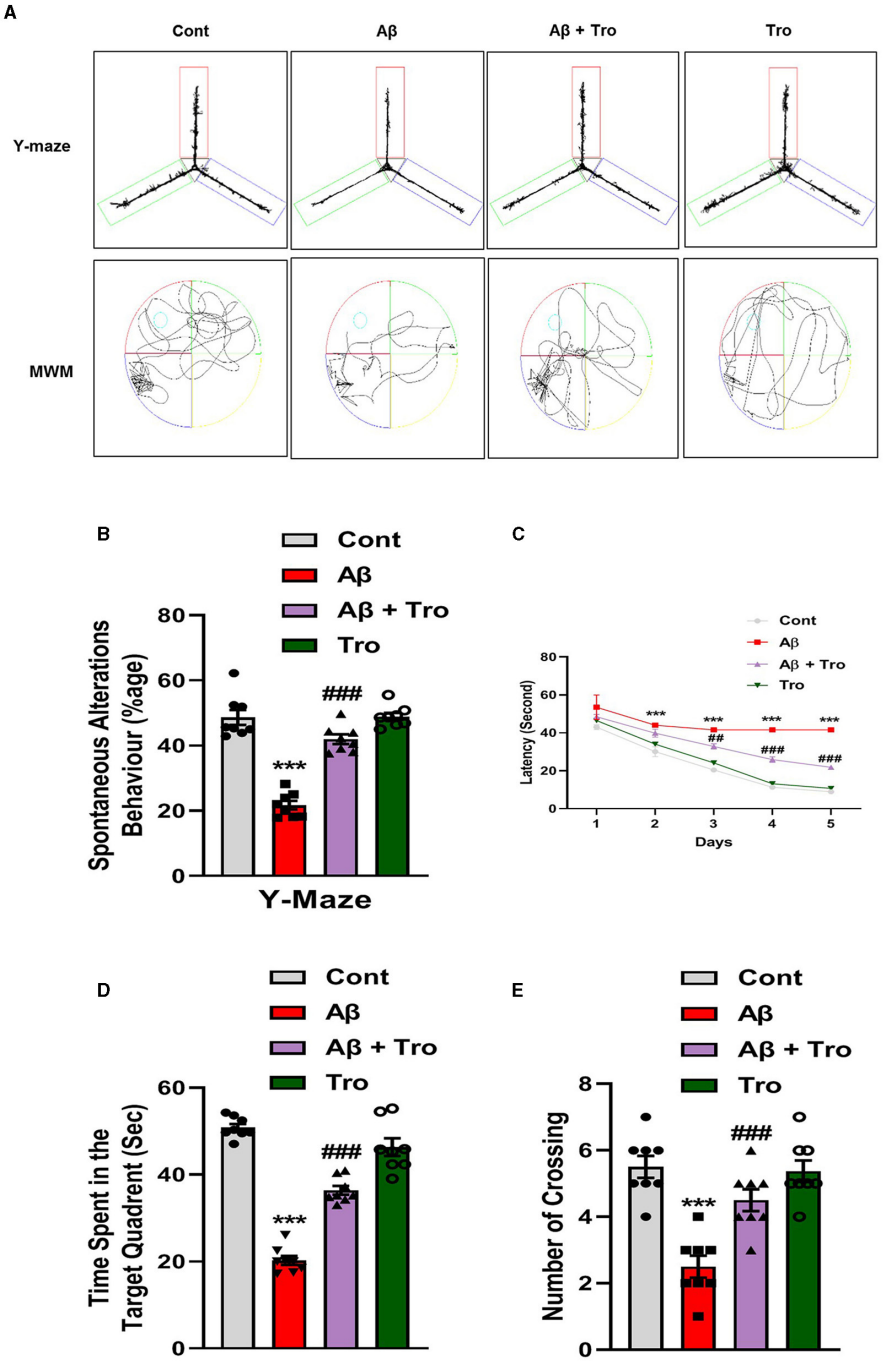
Neuroprotective effects of Trolox on memory and learning of mice brain. **(A)** The trajectory map of mice in Y-maze and MWM. **(B)** Y-maze test shows the percentage of spontaneous alteration behavior of experimental mice. **(C)** Line graph that represents average escape latency to reach the hidden platform until the fifth day. **(D)** Time spent by the mice in the quadrant where the platform was in training; **(E)** indicates the probe test. The data are presented as the mean ± SEM from eight mice per group, for Western blot and confocal microscopy, with four mice per group used in each assay, and are representative of three independent experiments. Scale bar = 50 μm, magnification 10×. Asterisks denote a significant difference from saline-injected control mice, and ^#^ indicates from Aβ_1 − 42_-injected mice. ***p < 0.001,^###^*p* < 0.001, and *p* < 0.05.

We performed neurobehavioral analysis (Y-maze and Morris water maze (MWM) tests) to examine the effects of Trolox on the memory and learning assessment of different experimental mice groups. First, we tested spatial working memory using the Y-maze (Figures 2A, B). Cognitive performance was assumed to be enhanced by a higher percentage (%) of spontaneous alteration behavior. Working memory was reduced in the Aβ_1 − 42_-injected mice group, presenting a significantly lower rate (21.68 %) of spontaneous alteration compared to the normal control mouse group (48.66 %) and Trolox-treated group (48.91 %), showing significant memory functions. Conversely, Trolox + Aβ_1 − 42_-treatment indicated a significant increase (41.97%) in spontaneous-alteration behavior compared with Aβ_1 − 42_-injected mice group, which demonstrated that Trolox mitigated short-term memory discrepancies in the Aβ_1 − 42_-injected mice group (Figures 2A, B). In the training phase of MWM, all groups' mice were allowed to search the hidden platform located in one quadrant of the water tank, which showed that AD mice took more time as compared to saline-treated control mice while treatment with Trolox reduced the time (Figure 2C). Furthermore, a probe test was performed after the training phase by removing the hidden platform. The results showed that Aβ_1 − 42_-induced mice spent less time and several crossings in a zone that previously had hidden platforms (Figures 2D, E). Time spent in the target quadrant and crossings over the hidden platform were significantly increased after treatment with Trolox. These results revealed that Trolox improved memory and learning functions.

### 4.2 Trolox-treatment downregulated the Aβ_1 − 42_, p-Tau and BACE1 expression causing Alzheimer's disease in mouse brain

**Figure 3:**
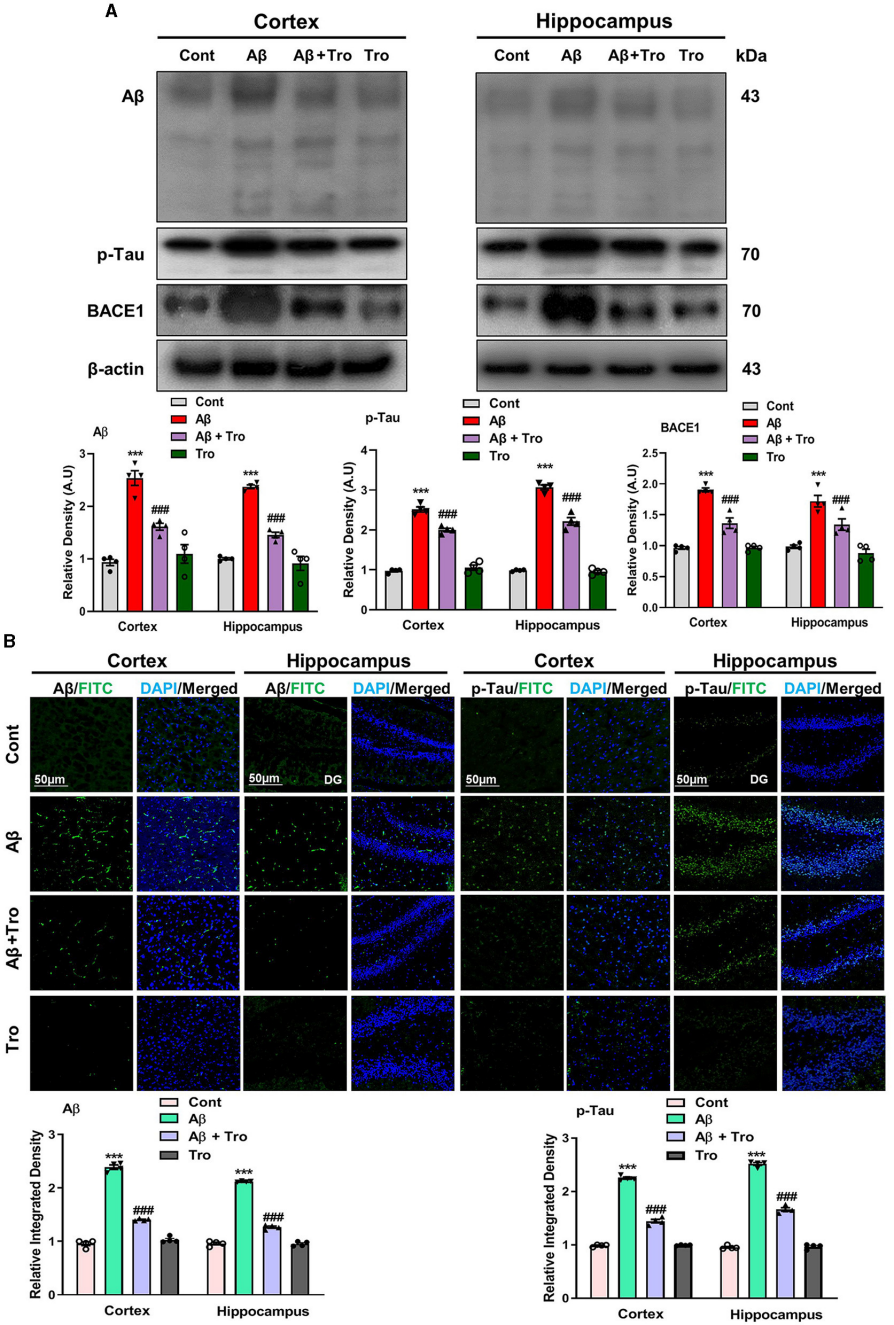
Trolox mitigated AD pathology by decreasing the Aβ, p-Tau, and BACE1 protein expressions. **(A)** Western blot analysis of Aβ, p-Tau, and BACE1 protein expressions in the cortex and hippocampus of mice brain. As a loading control, β-actin was used. Band quantification among the groups was performed using ImageJ software. **(B)** Confocal microscopy results show the immunofluorescence of Aβ and p-Tau (green) along with their respective histograms and DAPI staining (blue) in the cortex and hippocampus [dentate gyrus (DG)] of adult mice. The values of relative density compared with the control group are measured in arbitrary units (AU). The present data are measured as the mean ± SEM of eight mice/group, every four mice per group for Western blot and confocal microscopy, respectively, and are representative of three independent experiments. Scale bar = 50 μm, magnification 10×. Significance levels are indicated as ***p < 0.001 ^###^*p* < 0.001, and *p* < 0.05. Asterisks denote significant differences from saline-injected control mice, while hashtags indicate differences from Aβ_1 − 42_-injected mice.

According to the previous study, it has been investigated that a single dose of intracerebroventricular (i.c.v.) injection of Aβ_1 − 42_ peptides induces memory and cognitive impairment, causes deposition of Aβ and induces AD in healthy mice brains analogous to the indications detected in humans' brains (Khan et al., 2021; Liang et al., 2010). In line with these studies, our Western blot results exhibited higher expression for Aβ in the AD-induced mice's brain cortex and hippocampus than saline-treated control mice. Trolox treatment significantly reduced the expression of Aβ in the brain cortex and hippocampus in the AD mouse model (Figure 3A). Similarly, the tau protein is essential for stabilizing microtubules and plays a significant role in providing structural support and facilitating intracellular transport in neurons (Rawat et al., 2022). Our Western blot results demonstrated higher expression for p-Tau in both the cortex and hippocampus of AD-induced mice's brains as compared to saline-treated control mice. Interestingly, Trolox treatment significantly decreased the expression of p-Tau in the brain of AD-induced mice. β-secretase, or β-site amyloid precursor protein cleaving enzyme 1 (BACE1), plays an essential role in AD, which causes the formation of Aβ_1 − 42_ peptides by splitting the amyloid precursor protein (APP) (Mateos-Aparicio and Rodríguez-Moreno, 2019). Similarly, a higher expression level of BACE1 was found in both cortex and hippocampus in the brain of the Aβ_1 − 42_-injected mice group, which was reversed with the treatment of Trolox (Figure 3A). To further confirm the Aβ and p-Tau Western blot results, we performed confocal microscopy for the examination of immunoreactivity of Aβ and p-Tau in the AD-induced mice brain, which showed enhanced immunoreactivity of Aβ and p-Tau in the AD-induced mice brain compared to a control group of mice. At the same time, Trolox treatment effectively reduced the expression of Aβ in both the cortex and hippocampus (Figure 3B).

### 4.3 Trolox abrogated Aβ_1 − 42_-induced glial cell activation in AD mice brains

**Figure 4:**
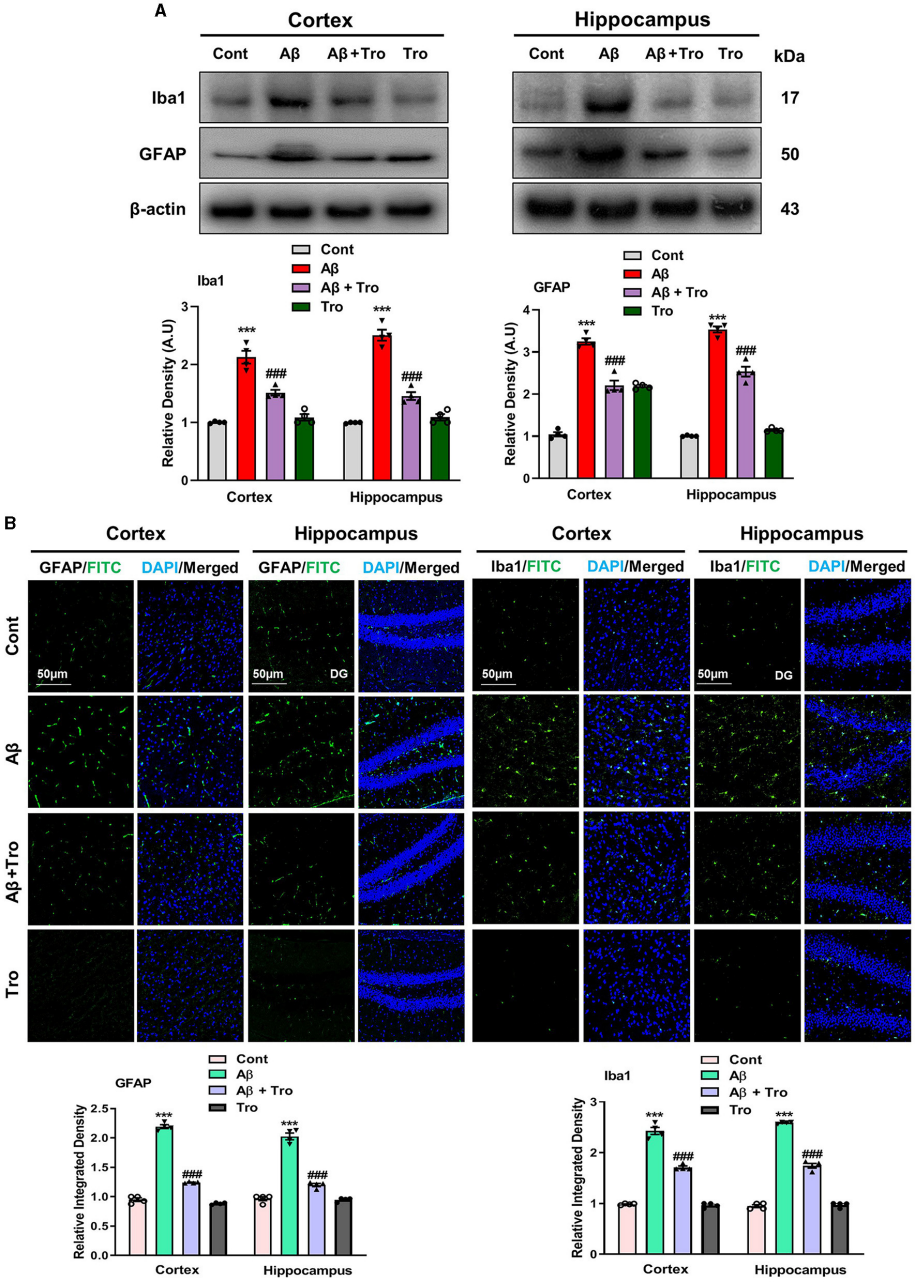
Effects of Trolox on reduction of glial cell activation. **(A)** Analysis of protein expression of Iba1 and GFAP in mice brain cortex and hippocampus, β-actin was used as a loading control. Bands were quantified among different mice groups by ImageJ software. **(B)** Confocal microscopy shows the immunoreactivity of GFAP and Iba1 (green) and DAPI staining (blue) in the cortex and hippocampus (DG) as well as their respective histograms. The values of relative density compared with the control group are measured in arbitrary units (AU). The data are presented as the mean ± SEM from eight mice per group for Western blot and confocal microscopy, with four mice per group used in each assay, and are representative of three independent experiments. Scale bar = 50 μm, magnification 10×. Significance levels are indicated as *** *p* < 0.001, ^###^*p* < 0.001, and *p* < 0.05. Asterisks (*) denote significant differences from saline-injected control mice, while hashtags (^#^) indicate differences from Aβ_1 − 42_-injected mice.

Within the brain, the glial cells (microglia and astrocytes) play a vital role in maintaining neuronal homeostasis. The accumulation of Aβ in the brain induces an excessive response from glial cells, triggering neuroinflammation and neurodegenerative (Kim et al., 2018). In our study, we examined the effects of Trolox in AD-induced activated glial cells, which showed higher expressions of Iba1 (Ionized calcium-binding adaptor molecule 1, a marker of activated microglia) and GFAP, (glial fibrillary acidic protein, a marker of activated astrocytes) in the AD-induced mice cortex and hippocampus as compared to saline-treated normal mice. At the same time, treatment with Trolox reduced the expression of activated glial cells (Figure 4A). For further confirmation of immunoblot results, we performed confocal microscopy to examine the immunoreactivity of GFAP and Iba1 in Aβ-induced mice brains. Immunofluorescence results revealed more immunoreactivity of GFAP and Iba1 in the cortex and hippocampus regions in the Aβ_1 − 42_ mice group compared with the control group. In contrast, co-administration of Trolox + Aβ_1 − 42_ significantly reduced the immunoreactivity of GFAP and Iba1 in the cortex and hippocampus regions in AD-induced mice (Figure 4B).

### 4.4 Trolox mitigated Aβ_1 − 42_-induced neuroinflammation by suppressing inflammatory cytokines in the AD mice brain

**Figure 5:**
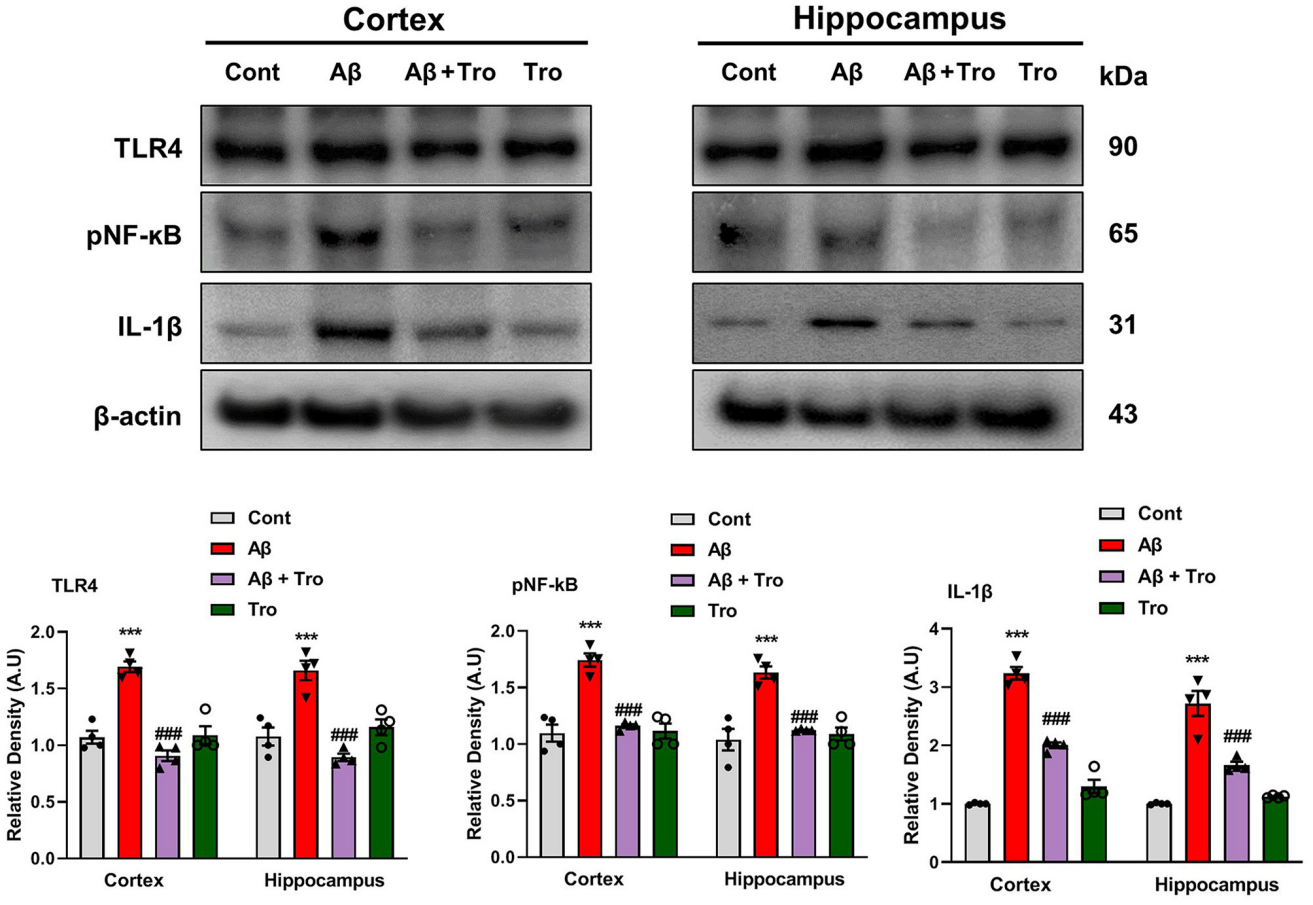
Trolox reduced inflammatory mediators in Aβ_1 − 42_-induced mice brains. Immunoblotting Analysis of TLR4, pNF-kB, and IL-1β proteins expressed in mice's brain cortex and hippocampus, β-actin was used as a loading control. ImageJ software was used to quantify bands across mouse groups along with their respective histograms. The values of relative density compared with the control group are measured in arbitrary units (AU). The data are presented as the mean ± SEM from eight mice per group for Western blot and confocal microscopy, with four mice per group used in each assay, and are representative of three independent experiments. Scale bar = 50 μm, magnification 10×. Significance levels are indicated as ***p < 0.001, ^###^*p* < 0.001, and *p* < 0.05. Asterisks denote significant differences from saline-injected control mice, while hashtags indicate differences from Aβ_1 − 42_-injected mice.

Toll-like receptor 4 (TLR4) is the surface receptor of glial cells (Iba1 and GFAP). We evaluated the effect of Trolox on these inflammatory cytokines (TLR4, pNF-kB, and IL-1β) using Western blotting. The expression of TLR4 is upregulated in the Aβ_1 − 42_ injected group, which was significantly downregulated in the Aβ_1 − 42_ + Trolox co-treated group compared to a control group of mice. We investigated the expression of phosphorylated nuclear factor kappa B (pNF-kB) in the experimental groups of mice. Since pNF-kB is crucial in the etiology of AD. Additionally, the interleukin 1β (IL-1β) is also released by the activated pNF-kB, which is responsible for neuroinflammation (Muhammad et al., 2019). The immunoblot analysis demonstrated the elevated expression of these biomarkers in both the cortex and hippocampus in the Aβ_1 − 42_-induced AD mouse model. Whereas Trolox treatment reduced the expressions of these inflammatory cytokines in the cortex and hippocampus of AD-induced mice brains (Figure 5). These investigations revealed that Trolox is a potent candidate for neuroinflammation.

### 4.5 Trolox suppressed the Aβ_1 − 42_-induced oxidative stress by elevating NRF2/HO1 level in AD mouse brain

**Figure 6:**
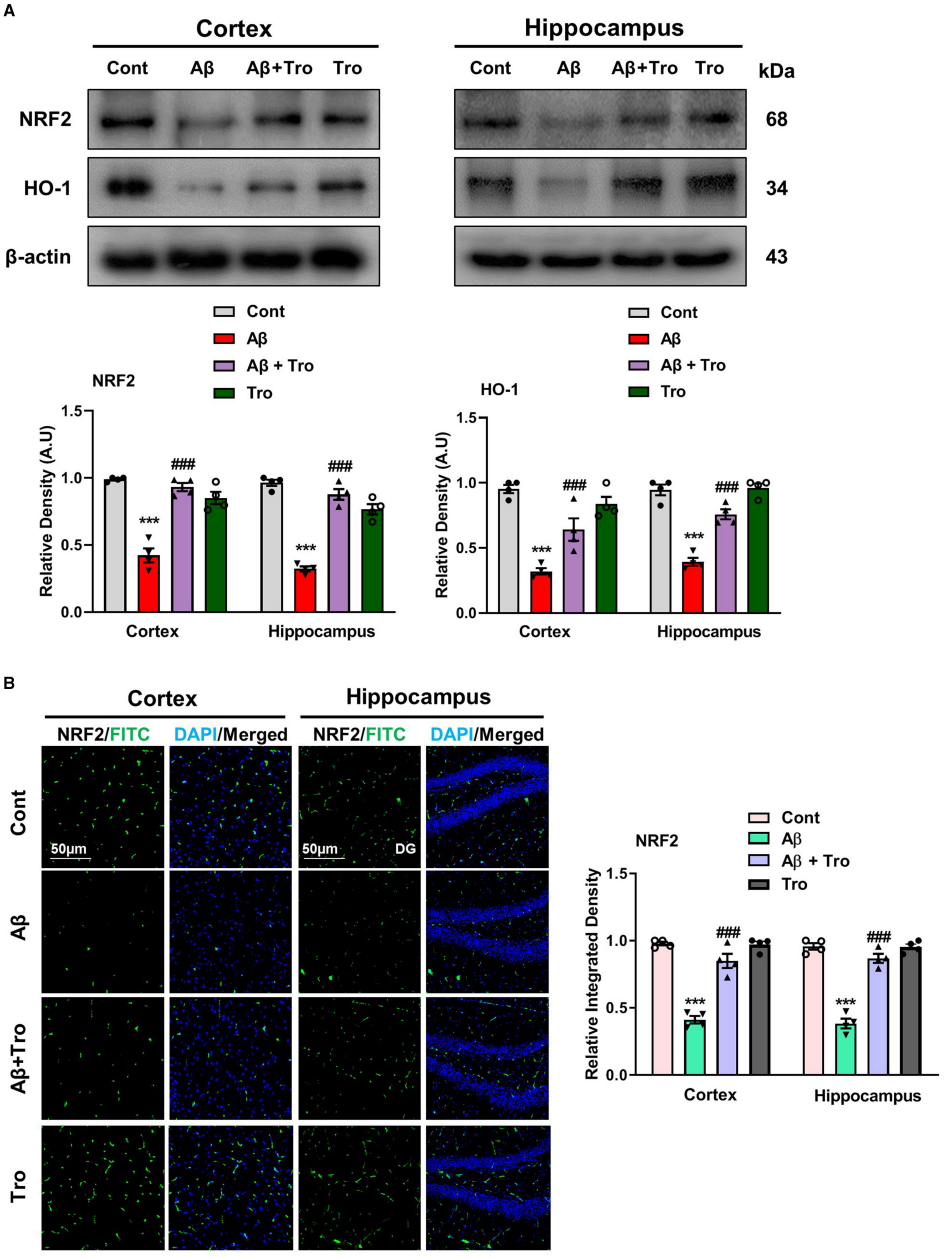
Neuroprotective effects of Trolox on oxidative stress. **(A)** Immunoblot analysis of the proteins NRF2 and HO-1 expression in cortex and hippocampus of adult mice brain. β-Actin as a loading control was used. **(B)** Immunofluorescence of NRF2 (green) along with their respective histograms and DAPI staining (blue) in both cortex and hippocampus (DG) of mice brain. The values of relative density compared with the control group are measured in arbitrary units (AU). The data are presented as the mean ± SEM from eight mice per group for Western blot and confocal microscopy, with four mice per group used in each assay, and are representative of three independent experiments. Magnification 10×, scale bar = 50 μm. Significance levels are indicated as ***p < 0.001, ^###^*p* < 0.001, and *p* < 0.05. Asterisks denote significant differences from saline-injected control mice, while hashtags indicate differences from Aβ_1 − 42_-injected mice.

Trolox has shown strong scavenging activity against various free radicals across multiple cellular model systems and has been approved as a reference compound as an antioxidant. The two eminent markers responsible for oxidative stress measurement are nuclear factor erythroid 2-related factor (NRF2) and heme oxygenase 1 (HO1), which perform defensive characteristics against oxidative stress in neurodegenerative conditions (Zhang et al., 2021). We also examined the antioxidative effects of Trolox against Aβ_1 − 42_-induced oxidative stress in the mouse brain cortex and hippocampus. Our Western blot analysis revealed significantly reduced levels of NRF2 and HO1 in both cortex and hippocampus in Aβ_1 − 42_-injected animals; surprisingly, Trolox treatment significantly enhanced the expression of NRF2/HO1 in the AD-induced mice brain (Figure 6A). We performed immunofluorescence analysis to confirm further our investigations, which showed a decreased level of NRF2 in the Aβ_1 − 42_-injected mice brains compared to saline-treated control mice and was upregulated with Trolox-treated mice brains (Figure 6B).

### 4.6 Trolox administration improved synaptic proteins in AD mouse brains

**Figure 7:**
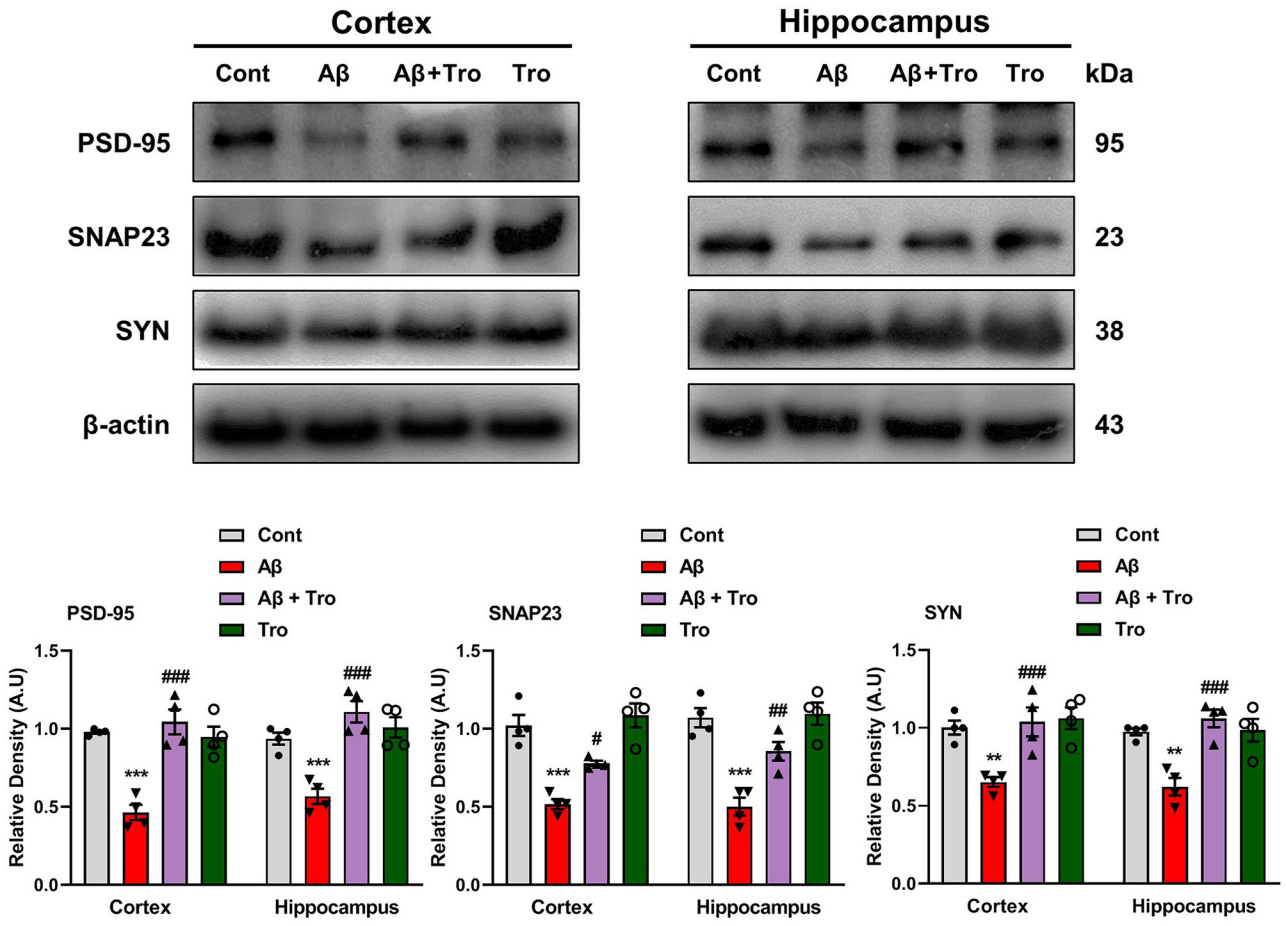
Trolox treatment enhanced the expression of synaptic proteins in the Aβ_1 − 42_-induced mice brain. Mouse cortex and hippocampus PSD-95, SNAP23, and SYP, protein expression analysis by Western blotting. β-actin was used as a loading control. Quantification of bands and histograms across mouse groups was carried out using ImageJ software. Compared to the control group, relative densities are expressed as arbitrary units (AU). The data are presented as the mean ± SEM from eight mice per group for Western blot and confocal microscopy, with four mice per group used in each assay, and are representative of three independent experiments. Scale bar = 50 μm, magnification 10×. Significance levels are indicated as ***p < 0.001, ^###^*p* < 0.001, and *p* < 0.05. Asterisks denote significant differences from saline-injected control mice, while hashtags indicate differences from Aβ_1 − 42_-injected mice.

It is reported that Aβ is toxic to synaptic function (Chen et al., 2014). For synaptic integrity analysis, we measured the synaptic proteins such as post-synaptic density protein 95 (PSD-95) and pre-synaptic proteins such as synaptosomal associated protein 23 (SNAP23) and synaptophysin (SYN). Our Western blot results showed reduced expression of these synaptic proteins compared to saline-treated control mice. On the contrary, Trolox treatment increases the expression levels of these synaptic markers in the AD mouse brain (Figure 7).

## 5 Discussion

**Figure 8:**
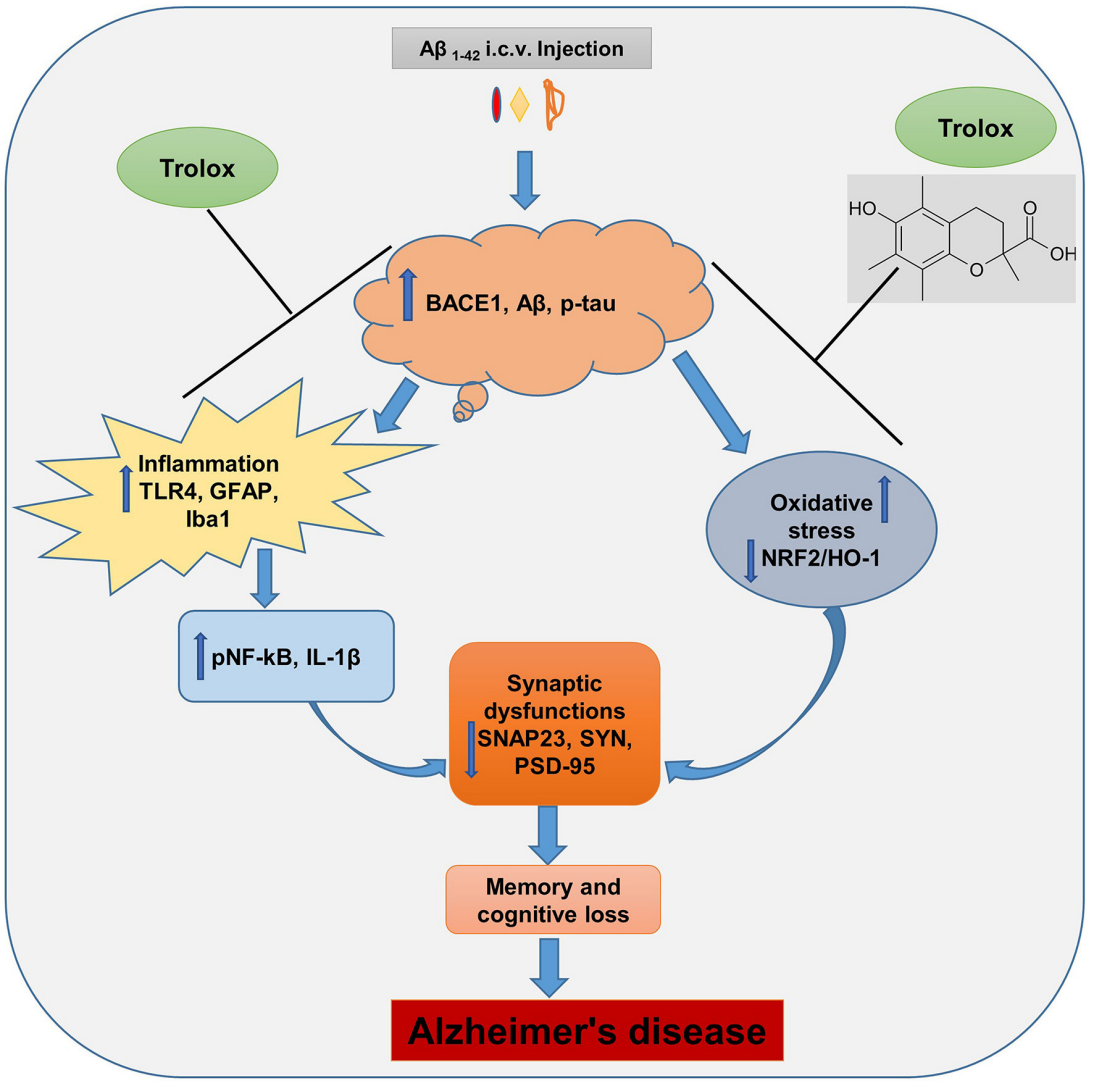
Probable mechanism of Trolox as a neuroprotective agent against Aβ_1 − 42_-induced AD.

The Aβ_1 − 42_ peptide synthesis and accumulation in the brain are the primary pathophysiological indicators of Alzheimer's disease (AD), which is the leading cause of dementia and neurodegenerative conditions (Ali et al., 2021). The Aβ accumulation in the brain induces neuroinflammation and oxidative stress, which further causes synaptic and memory dysfunction. Currently, numerous research studies are underway to lower the formation and content of Aβ in the brain of AD, and there is no proper treatment available for the patients. In this regard, therapeutic agents that showed anti-inflammatory and antioxidative properties were suitable candidates to reduce the inflammation and oxidative stress in the brain of AD patients. In our current study, we reported that Trolox possesses anti-inflammatory and antioxidant properties against Aβ_1 − 42_-induced Alzheimer's disease (AD) mouse model. Trolox reduced amyloid beta (Aβ) burden, neuroinflammation, and oxidative stress and enhanced synaptic memory and cognitive functions (Figure 8).

First, we examine the protective effects of Trolox in Aβ_1 − 42_-induced mice brains by performing behavioral tests, which showed that Trolox treatment in AD-induced mice significantly enhanced memory and cognitive functions. Tau protein is responsible for stabilizing microtubules to maintain the structural integrity of neurons. In AD conditions, tau protein is hyperphosphorylated, causing the formation of abnormal assemblies known as neurofibrillary tangles (NFTs). This hyperphosphorylation of tau protein causes it to separate from microtubules and then aggregate to form NFTs, which are related to neuronal dysfunction and cell death (Noble et al., 2013). Various studies have reported tau protein as a negative regulator in diseased conditions such as AD and Parkinson's disease. In AD and other tauopathies, tau is abnormally phosphorylated and results in neuronal and synaptic loss in the brain. Tau phosphorylation in the AD brain is considered by at least a three-fold increase in phosphorylation (Neddens et al., 2018). Our results showed upregulation of phosphorylated-tau (p-Tau) protein in both cortical and hippocampal regions of Aβ_1 − 42_-induced AD mice brain while Trolox treatment significantly reversed the p-tau expression as compared to the saline-treated mice group. Similarly, β-site amyloid precursor protein cleaving enzyme 1 (BACE1) is the vital enzyme that recruits the formation of Aβ from amyloid beta precursor protein (APP), leading to Aβ accumulation in the brain (Hajdú et al., 2023). Various conditions, such as AD hypoxia and oxidative stress, cause an increase in BACE1 expression (Cai et al., 2023). The findings of our current study exhibited elevated expression of BACE1 in the AD-induced mice. However, Trolox treatment significantly reduced the BACE1 expression.

The accumulation of Aβ in the brain is responsible for the activation of microglia and astrocytes, which further leads to neuroinflammation, oxidative stress, and neurodegeneration (Söllvander et al., 2016). We also observed a high expression level of ionized calcium-binding adaptor protein 1(Iba1), glial fibrillary acidic protein (GFAP), and other inflammatory mediators and cytokines (phosphorylated nuclear factor kappa B, interleukin 1β and Toll-like receptor 4) in AD-induced mice brains. In our current study Trolox treatment reduced the activated microglia and astrocytes and decreased the elevated inflammatory cytokines. On the other side, Aβ and neuroinflammation deteriorate the antioxidant enzymes in the brain, specifically the nuclear factor erythroid-related factor 2 (NFR2) and heme oxygenase 1 (HO1) pathway, which fights against oxidative damage. When this defense mechanism alters, it can harm brain cells, which further contributes to the progression of neurodegenerative conditions (Suzen et al., 2022). Our results showed that Trolox has strong antioxidative properties and prevents the mouse brain from oxidative stress by activating the NFR2/HO1 pathways.

Synaptic plasticity is important to the brain's development and normal functions, and its failure is a cardinal feature of AD (Mateos-Aparicio and Rodríguez-Moreno, 2019). The synaptic proteins such as post-synaptic density protein 95 (PSD-95), synaptophysin (SYN), and synaptosomal associated protein 23 (SNAP23) are essential for regulating various neurotransmitters and have been found to be modified in neurodegenerative diseases (Bereczki et al., 2018; Hsieh et al., 2006). In our study, we also found decreased expression levels of these synaptic proteins in Aβ_1 − 42_-induced AD mice brains, while the Trolox treatment significantly improved their expressions. In conclusion, our research findings revealed that Trolox exhibited multidimensional effects by reducing Aβ, p-tau, and BACE1 expressions both in cortical and hippocampal regions and mitigating memory, learning, and cognitive impairments in AD-induced mice. Further, it is demonstrated that Trolox has potent anti-inflammatory, antioxidative, and neuroprotective properties in AD mouse models. These findings underscore that Trolox is a promising therapeutic target for AD and related neurodegenerative disorders. However, further research studies should be needed to know the mechanistic role of Trolox in AD as well as clinical trials on humans in the future.

## Data Availability

The original contributions presented in the study are included in the article/Supplementary material, further inquiries can be directed to the corresponding author.
