# Supplementary material for: Multifaceted neuroprotective approach of Trolox in Alzheimer's disease mouse model: targeting Aβ pathology, neuroinflammation, oxidative stress, and synaptic dysfunction
Source: Front Cell Neurosci. 2024 Sep 17;18:1453038. doi: 10.3389/fncel.2024.1453038 (PMC11442280; doi:10.3389/fncel.2024.1453038)
Supplement: Supplementary file 1 [file Presentation_1.PPTX]

## Slide 1
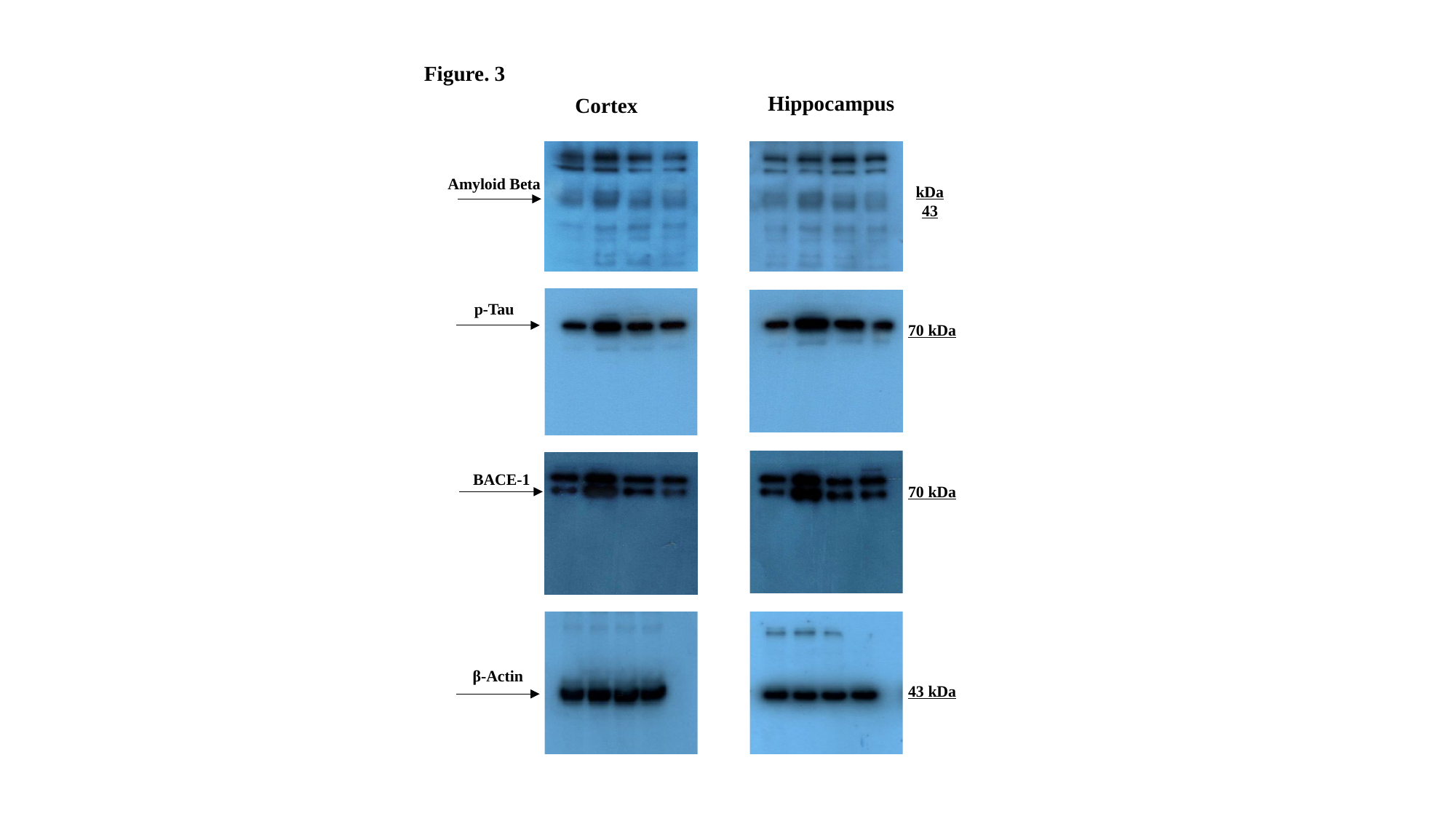

Figure. 3
Hippocampus
Cortex
Amyloid Beta
kDa
43
p-Tau
70 kDa
BACE-1
70 kDa
β-Actin
43 kDa

## Slide 2
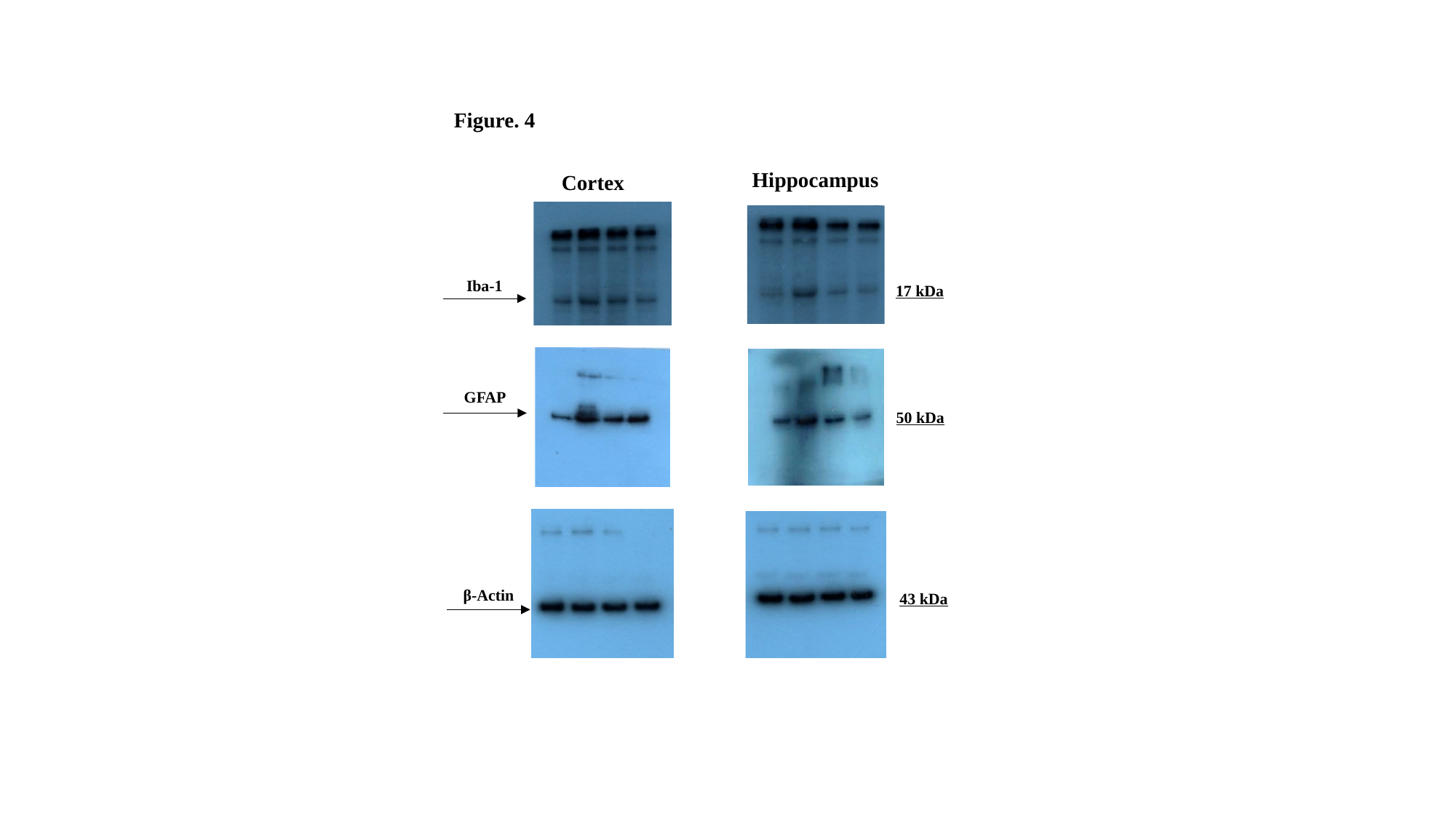

Figure. 4
Hippocampus
Cortex
Iba-1
17 kDa
GFAP
50 kDa
β-Actin
43 kDa

## Slide 3
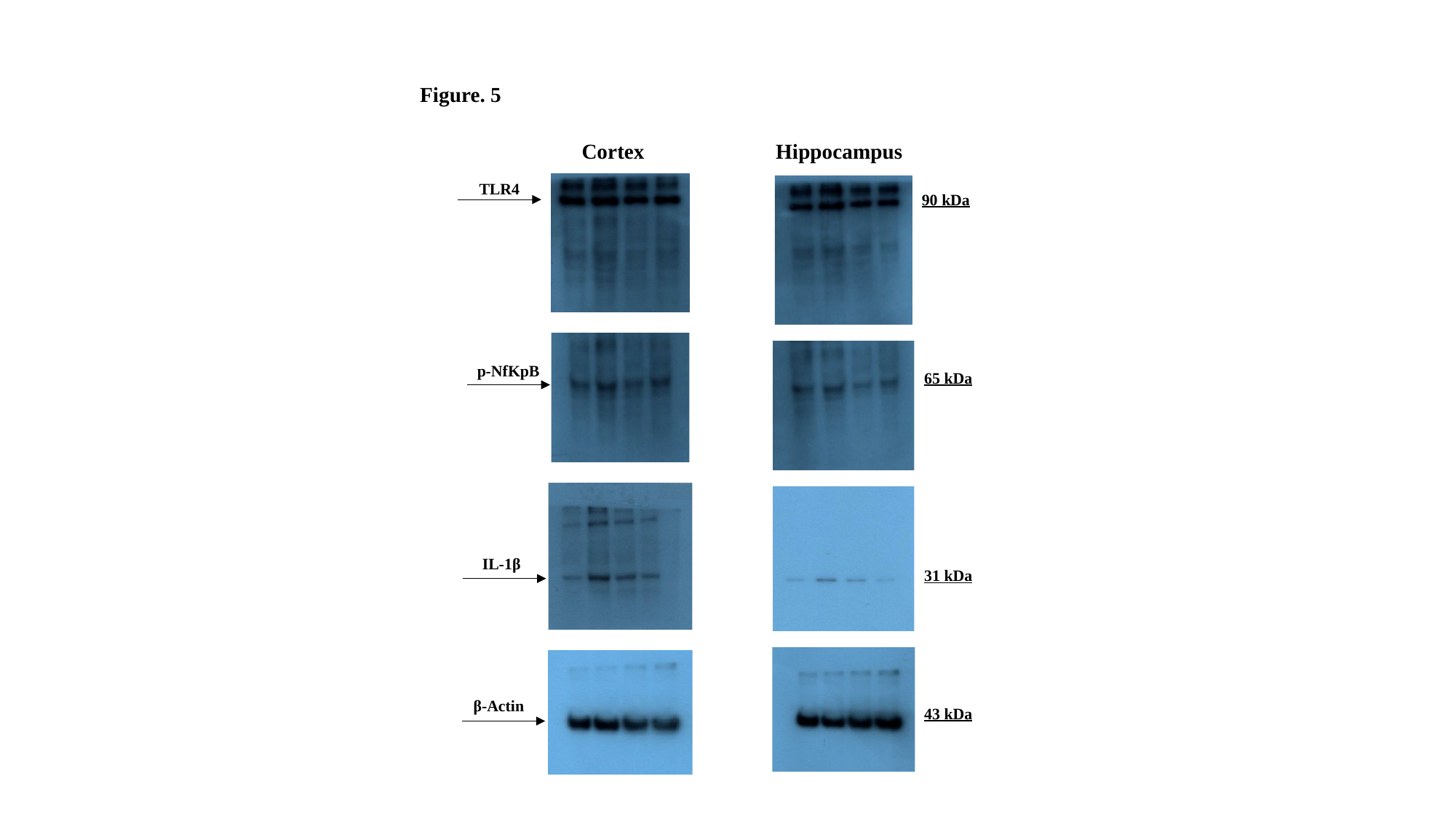

Figure. 5
Cortex
Hippocampus
TLR4
90 kDa
p-NfKpB
65 kDa
IL-1β
31 kDa
β-Actin
43 kDa

## Slide 4
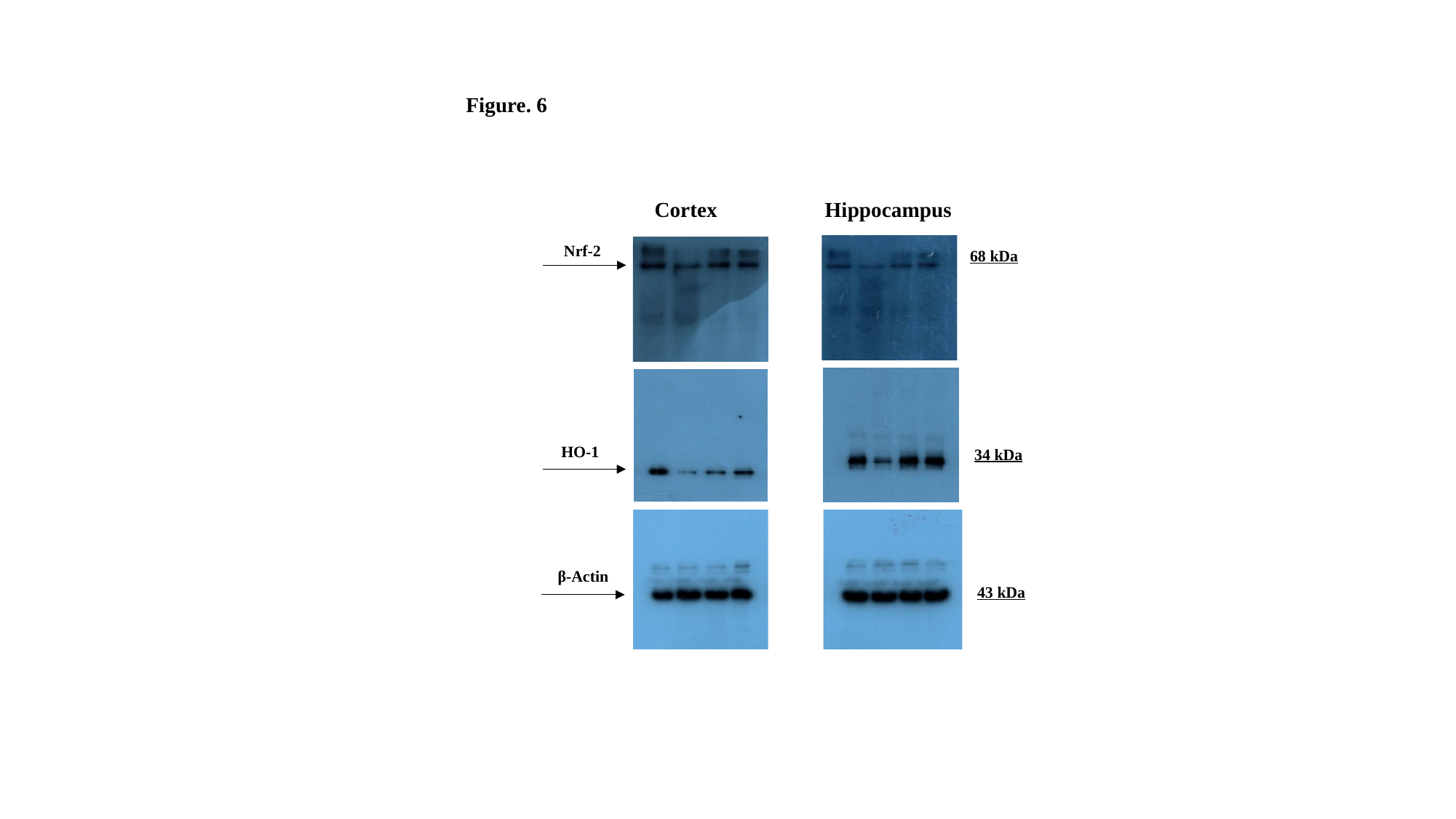

Figure. 6
Cortex
Hippocampus
Nrf-2
68 kDa
HO-1
34 kDa
β-Actin
43 kDa

## Slide 5
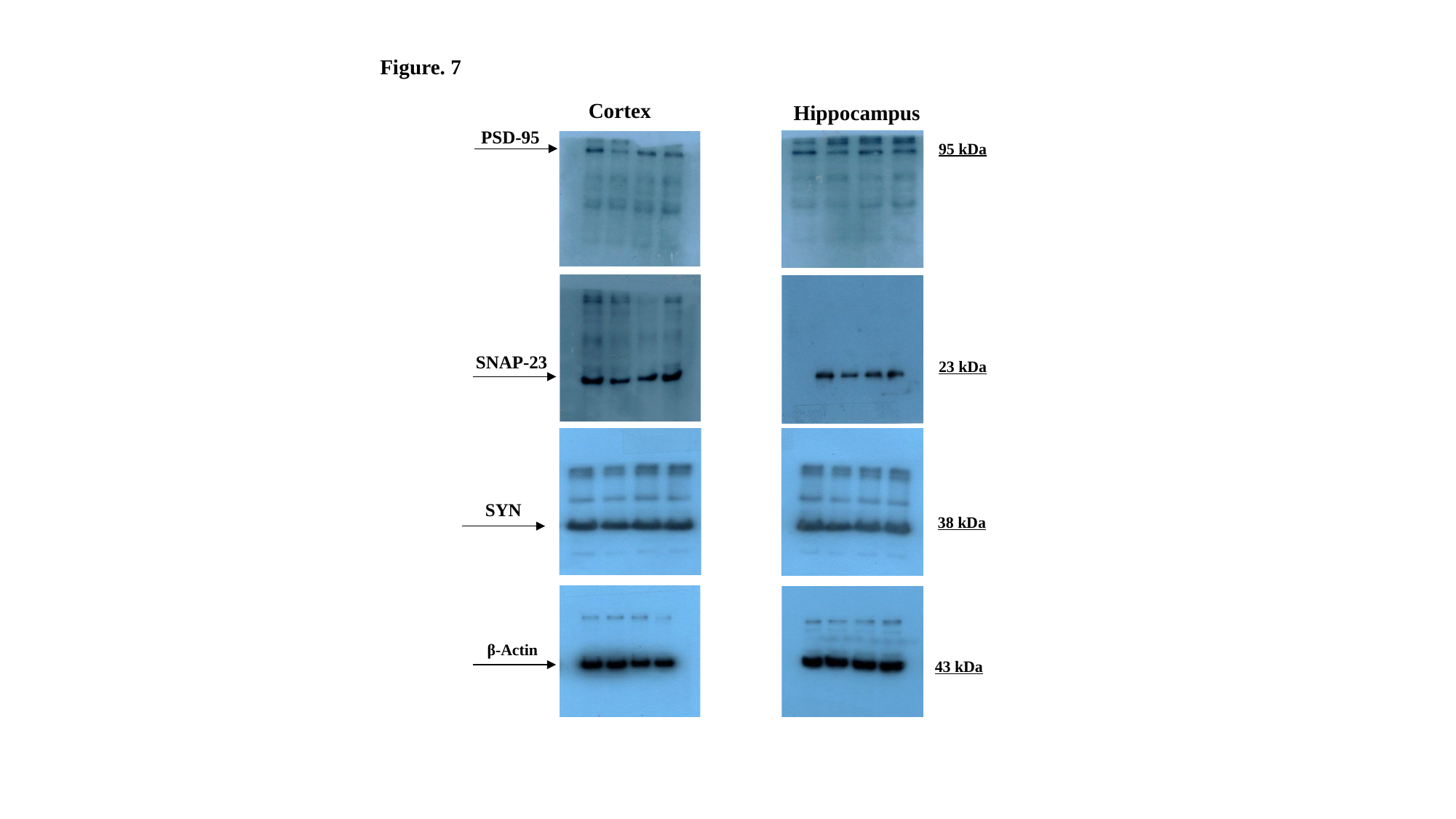

Figure. 7
Cortex
Hippocampus
PSD-95
95 kDa
SNAP-23
23 kDa
SYN
38 kDa
β-Actin
43 kDa
